# Unilateral Proptosis (Exophthalmos) Caused by Axillary-Subclavian Venous Thrombosis in a Patient with Upper Extremity Arteriovenous Dialysis Fistula

**DOI:** 10.14797/mdcvj.699

**Published:** 2021-10-20

**Authors:** Diego Gómez-Arbeláez, Ania García-Gutiérrez, José Antonio González-Fajardo

**Affiliations:** 1 Hospital Universitario 12 de Octubre, Madrid, Spain

**Keywords:** proptosis, exophthalmos, arteriovenous fistula, upper extremity deep venous thrombosis

## Abstract

Arterialization of orbital veins is most often due to dural arteriovenous malformations of the cavernous sinus area. We report an unusual case of unilateral proptosis (exophthalmos) caused by arterialized retrograde venous flow in the external jugular vein and cavernous sinus in a patient with an upper extremity hemodialysis fistula and ipsilateral acute central venous thrombosis. The patient’s symptoms improved after surgical closure of the hemodialysis fistula.

## Introduction

Arterialized blood flow in the cavernous sinus and ophthalmic veins (reversal of ophthalmic venous flow), and resultant abnormal venous drainage, ocular congestion, and hypertension can generate a wide variety of ophthalmic symptoms and signs, including proptosis or exophthalmos. Most often, this congestive phenomenon appears as a consequence of cavernous sinus area dural arteriovenous malformations.^1^ However, a similar clinical entity caused by arterialized retrograde venous flow in the jugular venous system also has been described.^2^,^3^,^4^,^5^ We report an unusual case of a patient with antecedent of left brachiocephalic arteriovenous hemodialysis fistula, and ipsilateral proptosis as clinical manifestation of acute axillary-subclavian venous thrombosis.

## Clinical Case

A 62-year-old man was admitted to our hospital with 3 weeks of left eye redness and ipsilateral hemifacial edema, as well as pain and edema of the left arm. The patient’s past medical history was significant for end-stage renal disease of nonestablished etiology, and his past surgical history was significant for surgical creation of left brachiocephalic arteriovenous hemodialysis fistula in 2011 and kidney transplant in 2012. The patient had no history of known prothrombotic disease or cancer. Prior to transplantation, the patient was dialyzed by a right central venous catheter (CVC), and the left upper extremity arteriovenous fistula (AVF) had never been used, as his kidney transplant had normal function.

On ophthalmological examination, visual acuity, visual fields, and extraocular movements of the left eye were normal. However, tortuous, dilated and hyperemic conjunctival vessels and proptosis were evident. On the left eye the intraocular pressure was moderately increased (26 mm Hg), and fundoscopy only revealed some arterio-venous crossing points bilaterally. Otherwise, the examination of the right eye was normal.

**Figure 1:**
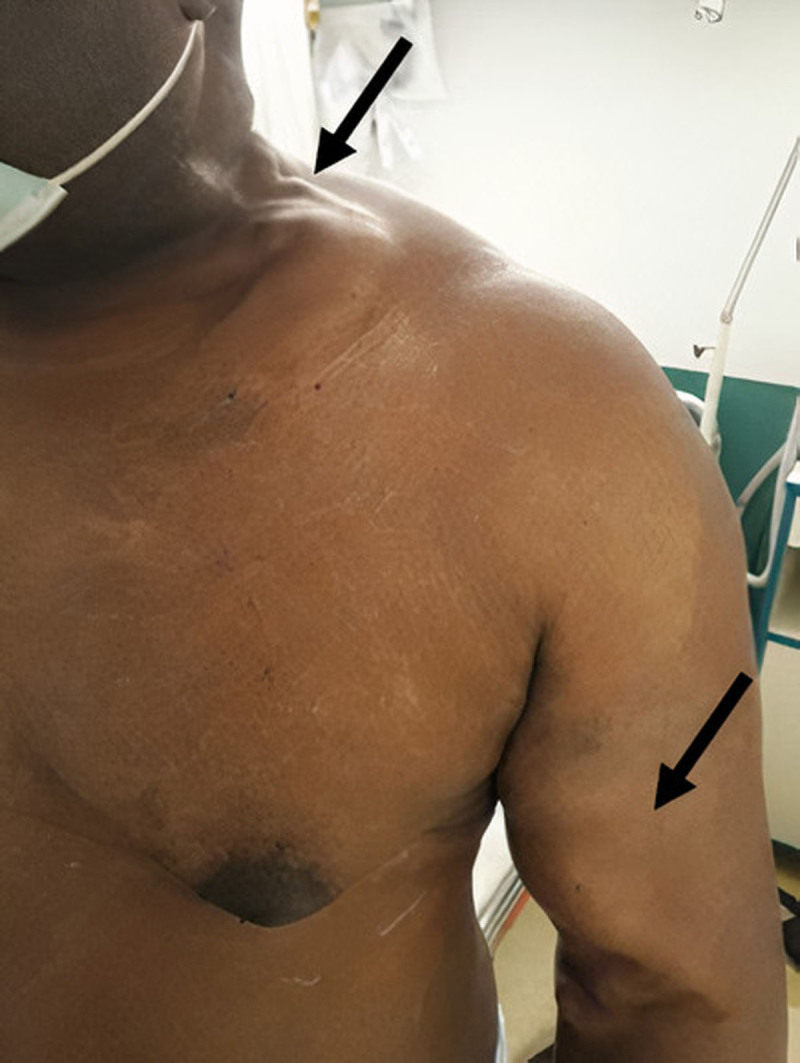
Aneurysmal brachiocephalic arteriovenous fistula and prominent axillosubclavian and external jugular veins in the left extremity.

**Figure 2:**
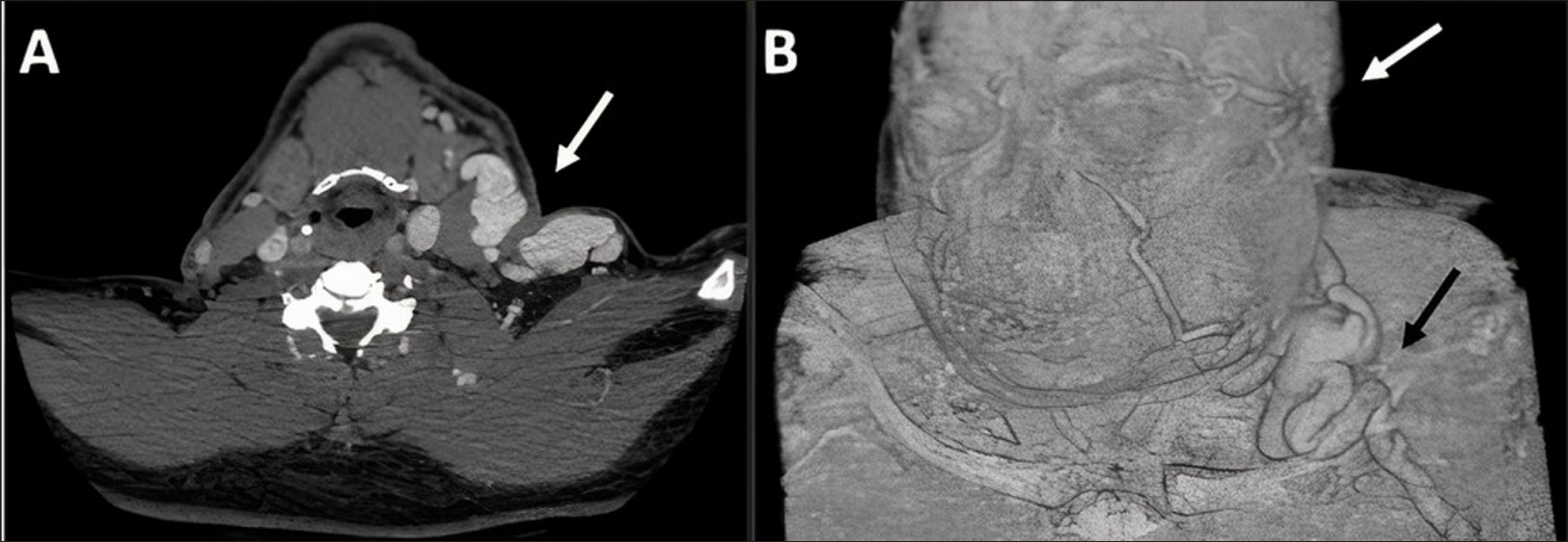
**(A)** Computed tomographic image shows retrograde dilatation of the left axillosubclavian and external jugular veins and **(B)** the venous drainage of the cranio-cervico-facial region.

**Figure 3:**
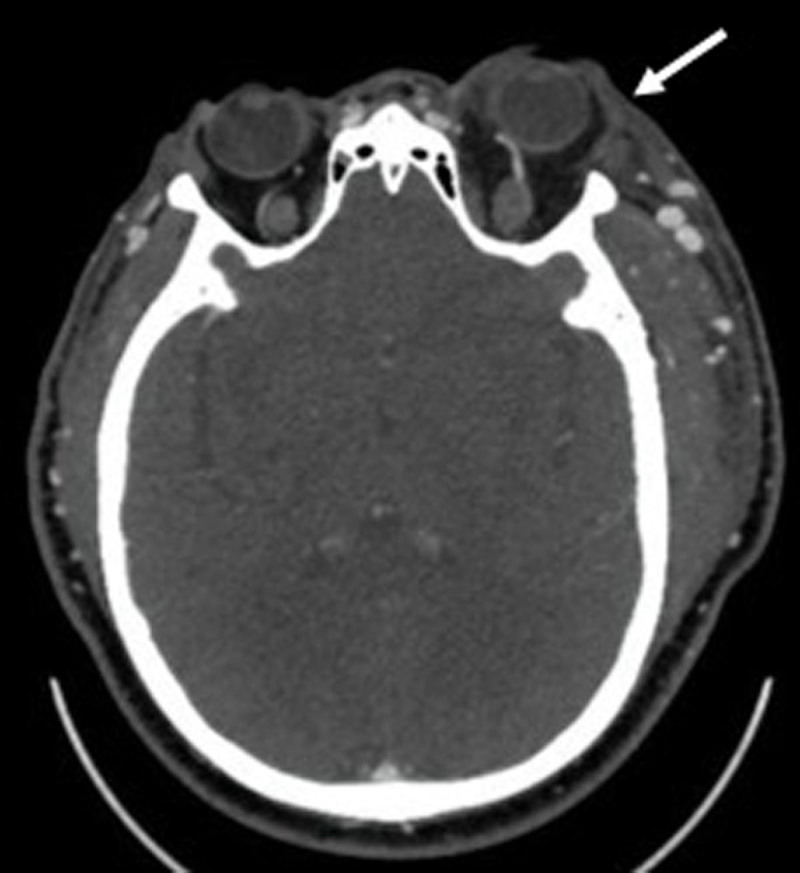
Computed tomographic image shows dilatation of the left superior ophthalmic vein and proptosis of the left eye in relation to an increased retrograde venous flow.

Physical examination revealed a large aneurysmal brachiocephalic AVF, and prominent deep veins over the upper arm and shoulder in the left extremity (***Figure 1***). Duplex ultrasonography showed acute thrombosis of the proximal segment of the left subclavian vein, patent AVF with flow volume of 800 mL/min, and blood return from the AVF travelling up in a retrograde fashion through the left external jugular vein. Computed tomography (CT) confirmed the central venous thrombosis and significant retrograde dilatation of the left axillosubclavian and external jugular veins (***Figure 2 A, B***), with retrograde flow in the cavernous sinus and superior ophthalmic vein and subsequent proptosis of the left eye (***Figure 3***). Laboratory findings were within normal limits, including a complete blood count, a coagulation profile, and comprehensive metabolic panel.

Therefore, the proptosis was attributable to retrograde blood flow in the left jugular venous system, originating from an upper extremity dialysis AVF in the presence of central venous occlusion. We confirmed that the AVF was a major contributor of the proptosis by occluding it with a blood pressure cuff, which rapidly improved the patient’s symptoms. Ligation of the brachiocephalic AVF was then performed, and after surgery the patient presented a striking improvement with a decrease in facial and upper limb edema, as well as complete disappearance of the proptosis. At discharge, oral anticoagulant treatment (acenocoumarol) was indicated for 6 months.

## Discussion

Arterialization of orbital veins, and its consequent abnormal ophthalmic venous drainage, may manifest clinically with a wide range of symptoms and signs, including subjective or audible bruits over the ocular globe, blurred vision, headache, diplopia, ocular pain, conjunctival injection, and proptosis.^1^ Frequently, this situation occurs secondary to carotid cavernous fistulas, dural venous fistulas, and cervical arteriovenous malformations.^1^ Here we report an unusual case of unilateral proptosis, conjunctival hyperemia, and hemifacial edema caused by arterialized retrograde venous flow in the external jugular vein, which is in relation to an ipsilateral upper extremity dialysis AVF in the presence of acute central venous thrombosis.

Several conditions, such as cancer, previous intimal trauma caused by CVC cannulation, inflammatory processes, immobility, and thrombophilias, may favor the occurrence of central venous thrombosis.^6^ Hypercoagulability associated with end-stage renal disease^7^ and elevated venous pressure caused by a hemodialysis AVF also have been described as contributors to the thrombotic process.^4^,^8^ Our patient had no history of prothrombotic disease or malignancy, renal graft function had remained stable since transplantation, and coagulation profile was unremarkable. Although the patient denies having had left-sided inserted central lines, the presence of prominent venous collaterals suggests the presence of a previous long-standing moderate- to high-grade stenosis that may have led to the thrombosis. Nonetheless, the exact underlying cause of central vein occlusion has not been fully elucidated. The patient’s proximal subclavian vein thrombosis, ipsilateral to the brachiocephalic arteriovenous hemodialysis fistula, redirected retrograde arterialized venous flow into the external jugular vein and the cavernous sinus. Unilateral proptosis was therefore caused by reversal of ophthalmic venous flow and raising the venous pressure along the outflow system of the eye.

Central venous thrombosis or stenosis usually has no clinical repercussions in the general population, but up to 70% of patients with an ipsilateral AVF may develop symptomatic venous hypertension.^9^ Ipsilateral arm swelling and high venous pressures during dialysis sessions have been described as the most common characteristics of symptomatic venous hypertension.^10^,^11^ Hemifacial swelling, visual disturbance, papilledema, and pulsatile tinnitus also have been reported.^5^,^12^,^13^ To our knowledge, only three previous cases of proptosis have been described in patients with central venous thrombosis or stenosis and an ipsilateral dialysis fistula.^2^,^4^

Recanalization of the occluded subclavian vein might be a therapeutic alternative, but we decided to discard this option due to suspicion of moderate- to high-grade chronic stenosis, which decreases the probability of success and increases the risk of rethrombosis. In addition, occlusion of the AVF with the sphygmomanometer showed a marked improvement in the patient’s symptoms. Hence, surgical closure of the hemodialysis fistula was chosen.

In conclusion, this case highlights that proptosis also may be caused by an extracranial vascular etiology. The possibility of central vein thrombosis should be considered in patients with a peripheral hemodialysis AVF and an ocular and facial congestive phenomenon. Timely diagnosis and treatment can prevent the onset of visual complications in the patients.
